# Intra-individual alterations of serum markers routinely used in forensic pathology depending on increasing post-mortem interval

**DOI:** 10.1038/s41598-018-31252-5

**Published:** 2018-08-24

**Authors:** Lina Woydt, Michael Bernhard, Holger Kirsten, Ralph Burkhardt, Niels Hammer, André Gries, Jan Dreßler, Benjamin Ondruschka

**Affiliations:** 1 Institute of Legal Medicine, Medical Faculty University of Leipzig, Leipzig, Germany; 2 Emergency Department, University Hospital of Leipzig, Leipzig, Germany; 3 Emergency Department, Heinrich Heine University Duesseldorf, Duesseldorf, Germany; 4 Institute for Medical Informatics, Statistics and Epidemiology, University of Leipzig, Leipzig, Germany; 5 LIFE Research Center for Civilization Diseases, University of Leipzig, Leipzig, Germany; 6 Institute of Laboratory Medicine, Clinical Chemistry and Molecular Diagnostics, University of Leipzig, Leipzig, Germany; 7 Department of Anatomy, University of Otago, Dunedin, New Zealand; 8 Department of Orthopedic and Trauma Surgery, University Hospital of Leipzig, Leipzig, Germany; 9 Fraunhofer IWU, Dresden, Germany

## Abstract

Post-mortem biochemistry of serum markers has been the subject of numerous studies, but *in-situ* marker stability after death has not been sufficiently evaluated yet. Such laboratory analyses are especially necessary in the cases of functional deaths without morphological evidence of the death causes and also in cardiac death cases with only very short survival times. The aim of the study was to determine the post-mortem stability of commonly-used serum markers at predefined time points. In 20 cases, peripheral venous samples were taken starting immediately after circulatory arrest and ending 48 hours after death. Serum creatinine, urea, 3-β-hydroxybutyrate, tryptase, myoglobin, troponin T, creatin kinase and creatin kinase-MB have been included. For all markers, we observed increasing marker levels for longer post-mortem intervals. Significant marker level changes began two hours after death. Excessive increases were observed for cardiac and muscle markers. Marker levels showed high intra-assay precision. Furthermore, the markers were robust enough to withstand freeze-thaw cycles. Potential contamination of arteriovenous blood did not influence the post-mortem marker levels. Post-mortem blood should be sampled as soon as possible, as increased post-mortem intervals may heavily change marker levels *in-situ* in individual cases, whereas the markers are mostly unaffected by laboratory conditions.

## Introduction

Since the 1960’s, numerous articles and intermittent reviews considering post-mortem biochemistry have been published^1–3^, showing both an increasing number of authors occupying themselves with this sector of forensic science and the relevance of this topic in legal medicine. This research has compiled publications regarding the retrieval of new markers, which show different causes of organic or functional death circumstances and their relation and measurement methods in different body fluids. Also included are proposals considering threshold values (‘cut-offs’) of markers dependent on causes of death and establishment of advanced methodology, with the aim of creating fast and cheap next-to-dissection table-tests.

Nevertheless, the application of biochemical markers in post-mortem investigations of both disciplines, forensic and clinical pathology, is still not well-established and is not used routinely, although biochemistry is declared necessary to solve the ultimate causes of death in about 10% of natural deaths in forensic routine^3^, e.g. in cases of acute kidney failure, metabolic ketoacidosis, anaphylaxis and in cardiac death cases with only very short survival times. A lack of reliable data may be a problem leading to uncertainty about using clinical chemistry methods^4,5^. Additionally, an interpretation of post-mortem laboratory results of blood samples gets progressively more difficult with proceeding hemolysis, which is why alternative body fluids such as cerebrospinal fluid, urine, pericardial fluid and especially vitreous humour are increasingly being used^3,6,7^.

However, there are, unrelated to the cause of death, hardly any published research attempts investigating the intra-individual stability of markers used in forensic medicine by serial peripheral venous blood measurements in the same body after the onset of death. Very few existing studies address the small number of biochemical markers of sepsis and anaphylaxis^8–12^, and myoglobin^13^ in post-mortem serum; there remain many organ systems whose characteristic markers have not been evaluated considering intra-individual changes after death.

This study involved a number of serum markers, which are well established for the clinical routine in living patients and are becoming increasingly important in the forensic setting as well. In this study, the biomarkers creatinine and urea are markers of renal insufficiency, 3-β-hydroxybutyrate (3HB) is the target marker for alcoholic and diabetic ketoacidosis and tryptase is a parameter of mast cell degranulation and indicative for anaphylaxis and other allergic conditions. Myoglobin, troponin T (with a high-sensitive assay), creatin kinase (CK) and CK-MB are markers for cardiac and/or coronary diseases. All were measured repeatedly in blood samples of the bodies post-mortem to determine their intra-individual *in-situ* stability after death in predefined time intervals and are outlined briefly in the following paragraph.

Typical renal markers in clinical and forensic settings are creatinine and urea. Creatinine is generated in skeletal muscle by dehydration of phosphocreatine. Creatinine is mainly eliminated from circulation by glomerular filtration and its blood concentration is an established marker of kidney function. Similarly, urea is considered an end product of protein metabolism, and is eliminated almost exclusively by the kidneys in urine. For both renal markers, elevated post-mortem blood levels can be found in cases of renal or skeletal muscle damage^14–16^, thereby forming an important marker in the forensic routine.

3HB together with acetone and acetoacetate forms the group of ketone bodies. It is synthesized hepatically and forms an important serum marker in the case of pathologic situations of carbohydrate deficiency. Here it provides an alternative energetic source for the organs^17,18^, especially for the brain. Common conditions of elevated ketone body levels in the clinical routine are alcoholic or diabetic ketoacidosis^17,19,20^. Equally, 3HB forms an important marker in the forensic routine for ketoacidosis.

Tryptase is a serine protease secreted by mast cells. The numerous functions of tryptase are not completely clear, but it is a highly specific marker for mast cell degranulation and therefore increases in conditions of immediate allergic reactions^21,22^. In the context of forensic medicine, post-mortem serum tryptase has been established as an indicator of possible ante-mortem anaphylaxis^22^.

Myoglobin can be elevated due to several causes^13,23^. Myoglobin is an oxygen-binding protein found in the heart and in skeletal muscle and is consequently used most often to detect myocardial and skeletal muscle damage in clinical medicine. Troponin T is a regulatory protein found exclusively in heart muscle cells, and is released into the blood from injured cardiac myocytes about three hours after ischemic injury. Troponin T increases in many diseases^24^. CK is a kinase mainly situated in muscle tissue and plays an important role in cellular energy generation through a process of phosphorylation. The isoenzyme CK-MB exists notably in cardiac myocytes and is therefore a more specific cardiac marker^25^.

The aim of the present study was to show if a selection of widely known and used biochemical markers of renal insufficiency, ketoacidosis, anaphylaxis or ischemic heart damage could be declared as stable intra-individually in the early post-mortem interval to conclude their reliability in forensic application.

## Results

### Demographic and autopsy data

The study included six females and fourteen males, whose ages at death varied between 29 to 98 years (median age 70.5 years, interquartile range (IQR) 23.5 years). 18 patients were resuscitated before the final declaration of death, and two patients were not. The duration of cardiopulmonary resuscitation (CPR), if performed, varied between 10 to 160 minutes (median 68.5 minutes, IQR 46.5 minutes).

**Figure 1:**
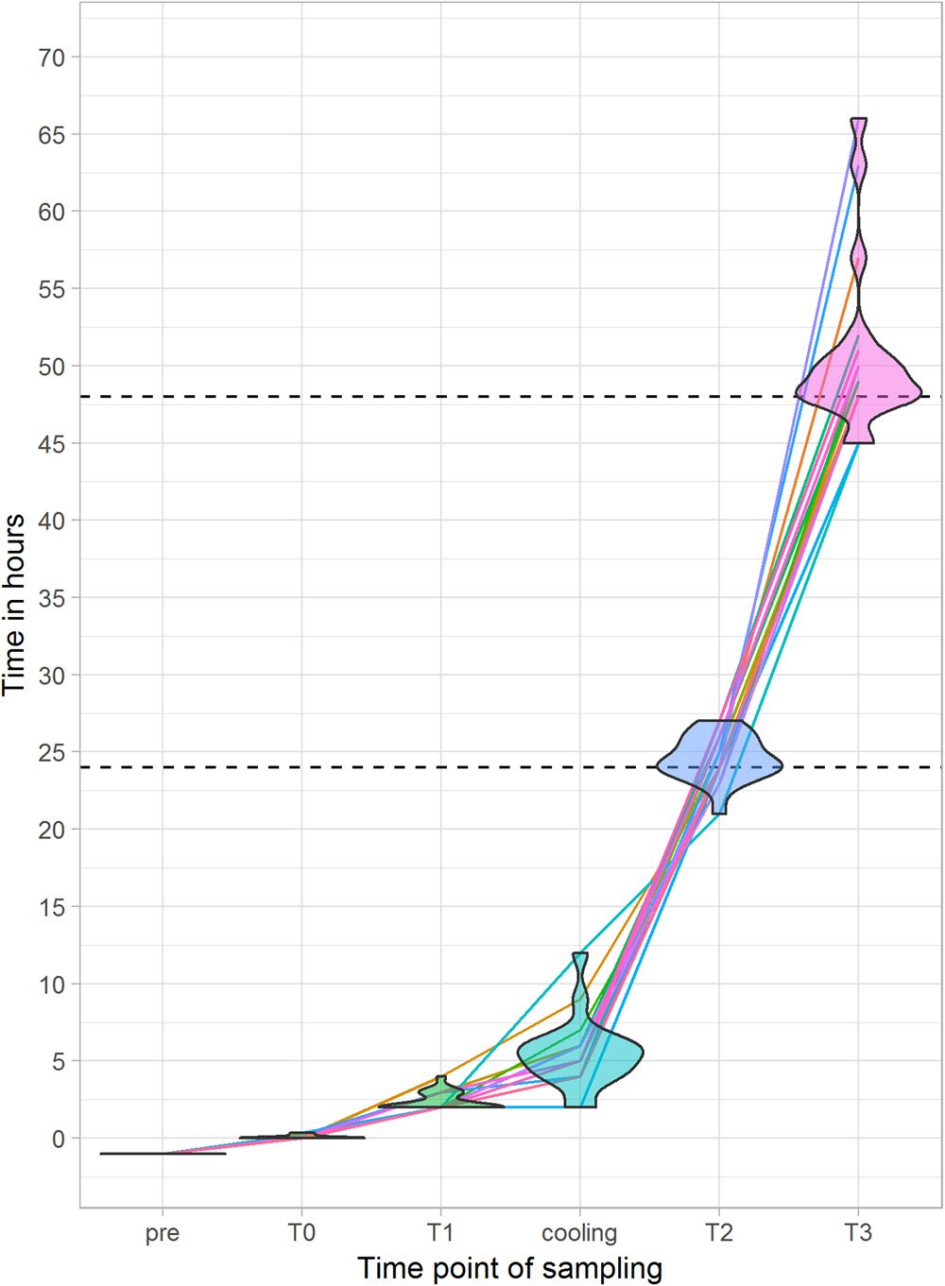
Distribution of single sampling over time after death, where T0 is defined as the moment of death. ‘Pre’ means clinical sampling during resuscitation attempts by emergency doctors.

The different time points of blood sampling for this study are illustrated in Fig. 1. We collected blood immediately after death (=T0), after two hours (=T1), after one day (=T2) and after two days (=T3) post-mortem.

Autopsy was ordered by the public prosecutor’s office in half of all cases. In eight cases, autopsy revealed a cardiac cause of death (acute myocardial infarction, n = 6; congestive heart failure, n = 2). In two cases death was due to pulmonary causes (pulmonary thromboembolism, n = 1; aspiration of stomach content, n = 1). The ten cases, which were not ordered for autopsy, were classified as formally unknown, but none of them showed severe injuries during external examination.

Out of these 10 cases without final diagnosis, the emergency doctors raised suspicion of four cardiac causes of death (acute myocardial infarction, n = 1; congestive heart failure, n = 2; sudden cardiac death, n = 1), two pulmonary causes (pulmonary thromboembolism, n = 2), aortic dissection type a (n = 1), hemorrhagic shock due to gastrointestinal bleeding (n = 1) and acute ischemia of the intestines (n = 1) as plausible causes of death, while one case remained completely uncertain.

Marker levels were checked for any confounding effects by age and gender of the deceased. The age did not show any significant interaction between the measured values (all p > 0.05). Urea levels were higher in females than males (p < 0.05). All other markers did not show gender-dependent distribution.

### Sample indices

The serum samples showed increasing grades of hemolysis and lipemia correlating with the length of the post-mortem interval (each p < 0.001), whereas the icterus index of the samples remained stable over the tested time period (p = 0.137) with the tendency to decline (please see Supplemental Figs. 1 and 2).

Table 1 shows overall comparisons between changes in sample indices and marker levels. The marker levels depend on the severity of post-mortem blood changes related to altered hemolysis and lipemia content. Furthermore, the marker levels were independent to the icterus index of the samples.

**Table 1:** Comparison between different sample indices and corresponding marker levels.

Marker	Hemolysis index		Lipemia index		Icterus index	
	Standard error	*P* value	Standard error	*P* value	Standard error	*P* value
Creatinine	0.039	<0.001	0.044	<0.001	0.107	0.953
Urea	0.018	<0.001	0.020	<0.001	0.059	0.239
3HB	0.041	0.042	0.052	<0.001	0.115	0.283
Tryptase	0.039	<0.001	0.053	<0.001	0.135	0.820
Myoglobin	0.158	<0.001	0.269	<0.001	0.374	0.810
Troponin T	0.135	0.018	0.198	0.007	0.358	0.463
CK	0.209	<0.001	0.330	<0.001	0.511	0.743
CK-MB	0.144	<0.001	0.231	<0.001	0.372	0.888

Significance was tested with a linear mixed regression model by modelling individuals as random intercept term. P values were adjusted according to the Benjamini-Hochberg procedure. 3HB, 3-β-hydroxybutyrate; CK, creatin kinase.

### Serum levels of analytes according to post-mortem interval

**Figure 2:**
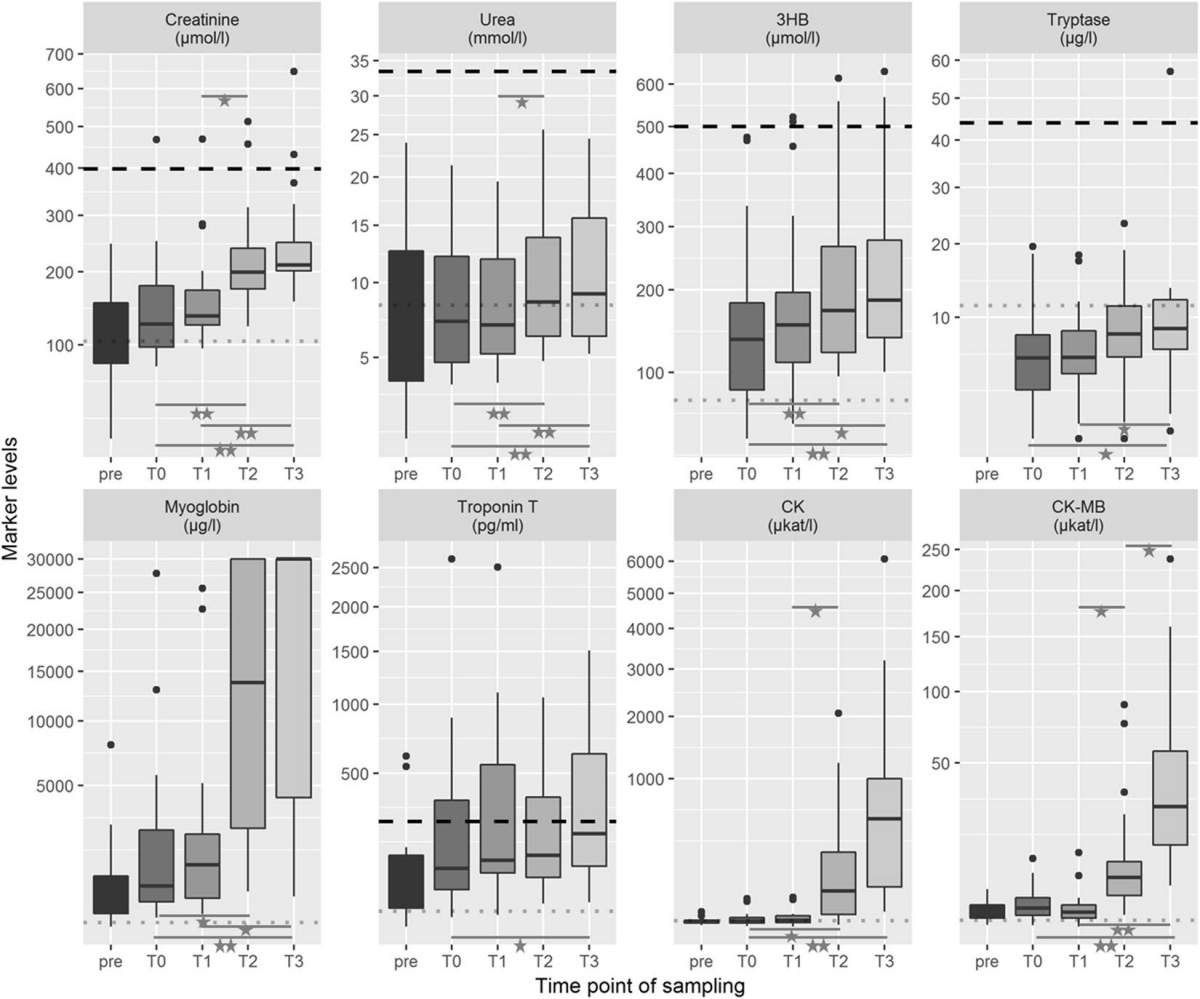
Box plot diagrams for the biochemical markers tested, listed by their time point of sampling (T0 defined as moment of death). The outlines of the boxes indicate the 25% and 75% percentile, the solid black line the median. End of lines show the minima and maxima. Outliers (>1.5 times interquartile range) were presented as bold points. Values outside the measurement ranges (detected for tryptase, n = 2; myoglobin, n = 17; and troponin T, n = 2) were defined as maximum or minimum of the limits. For better visualization, troponin T values > 20,000 pg/ml (n = 2) were not illustrated in the diagram but were included in all statistical calculations. ‘Pre’ means clinical sampling during resuscitation attempts by emergency doctors. The dotted thin line in grey illustrate the clinical reference value, whereas the dashed thick line in black represents published post-mortem cut-off values (for references see text). *p < 0.05; **p < 0.001 using Friedman test followed by *post-hoc* Conover and Bonferroni adjustment. 3HB, 3-β-hydroxybutyrate; CK, creatin kinase.

The development of every marker is visualized as box plot (see Fig. 2). The median values and their IQRs for all sampling times are shown in Table 2. In summary, median marker levels increased with longer post-mortem intervals starting at their lowest post-mortem measurement points with T0 and ending with their highest medians at T3 (p < 0.001). Whenever available, the T0 levels were approximately equivalent to the final laboratory measurement during the lifetime of the patients (ante-mortem data were archived for renal and cardiac markers). Quantification of marker levels at T3 showed significantly higher median values for all markers tested compared to T0. No significant changes were observed for any marker in the first hours after death (between T0 and T1). Tryptase, troponin T and CK-MB showed no relevant concentration changes in the first day after death (between T0 and T2).

**Table 2:** Median values and interquartile ranges (IQR) of the eight different markers for all sampling times (in hours post-mortem, hpm).

Marker	Pre(pre-final)	T0(death)	T1(2 hpm)	T2(24 hpm)	T3(49 hpm)
**Creatinine (µmol/l)**					
Median	100.0	125.0	135.5	199.5	211.0
IQR	73.3	80.8	47.8	65.3	48.5
**Urea (mmol/l)**					
Median	5.4	7.2	7.0	8.6	9.2
IQR	8.9	7.5	6.7	7.7	9.5
**3HB (µmol/l)**					
Median	n.m.	135.9	153.5	172.1	185.9
IQR	n.m.	99.8	87.0	146.2	139.0
**Tryptase (µg/l)**					
Median	n.m.	6.0	6.0	8.3	8.8
IQR	n.m.	4.7	3.9	5.3	5.4
**Myoglobin (µg/l)**					
Median	323.5	640.1	1,206	13,775	30,000
IQR	714.8	2,216	1,971	27,351	25,579
**Troponin T (pg/ml)**					
Median	53.4	90.5	113.4	134.6	213.5
IQR	110.6	305.6	471.9	400.6	622.3
**CK (µkat/l)**					
Median	2.5	2.6	3.3	64.1	535.8
IQR	1.7	4.0	5.7	250.9	922.0
**CK-MB (µkat/l)**					
Median	1.3	1.3	0.9	5.7	28.0
IQR	0.8	1.7	1.0	6.5	46.1

‘Pre’ means clinical sampling during final minutes ante-mortem. 3HB, 3-β-hydroxybutyrate; CK, creatin kinase; n.m., no measurement.

There were single cases with slight decreases in marker levels over time. One case with acute myocardial infarction as cause of death showed the highest initial creatinine value (467 µmol/l) with mild post-mortem decrease to T3 (431 µmol/l). In only two cases, a mild post-mortem decrease from T0 to T3 was shown for 3HB (136.7 to 115 µmol/l and 278.5 to 231.2 µmol/l). Further, two cases showed minute decreases of tryptase with longer post-mortem intervals (19.6 to 13.6 µg/l and 6.8 to 5.3 µg/l).

Cardiac markers showed controversial individual behavior. More than half of the cases (13/20 = 65.0%) showed myoglobin levels surpassing the upper measurement limit of 30,000 µg/l at T2, T3 or at both time points. However, there were three cases with decreasing myoglobin levels between T0 and T3. Troponin T reached its maximum detection limit of the test kit once at T3. Again, there were also five cases (5/20 = 25.0%) with decreasing values. Both CK and CK-MB showed unidirectional increases over time only.

### Marker concentrations and reference values

All measurements were performed using *in-vitro* diagnostics certified assays. In laboratory diagnostics, established reference intervals are applied to identify abnormal results, allowing a differentiation between ‘case/ill’ or ‘control/healthy’ (see Supplemental Table 1). Almost all post-mortem values of creatinine, 3HB, myoglobin and troponin T as well as all T2 and T3 levels of CK and CK-MB were higher than clinical reference ranges. Most of the samples showed urea and tryptase levels within their clinical reference spectrum.

**Figure 3:**
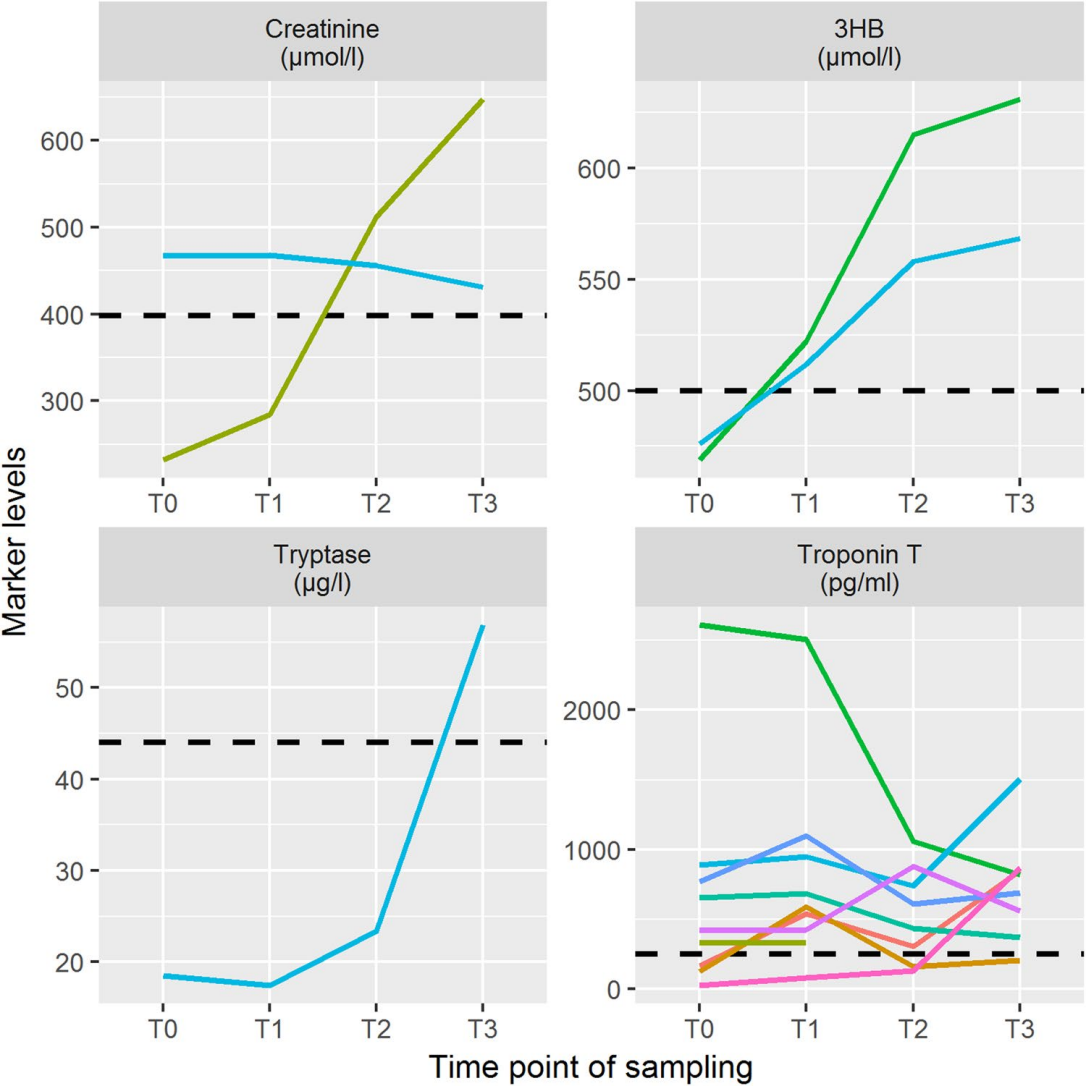
Measurement results for single cases with marker values above post-mortem cut-off values for creatinine, 3-β-hydroxybutyrate (3HB), tryptase and troponin T.

When using predefined post-mortem references, which were available for creatinine and urea^26^, 3HB^27^, tryptase^28^ and troponin T^29^, only single cases exceeded these cut-off values in the time course. All cases with at least one single measurement above these post-mortem thresholds are depicted in Fig. 3. Of interest, no single measurement exceeded the critical urea level (33.4 mmol/l; higher levels are indicative for a fatal acute kidney failure). The proposed cut-off values were not reached for creatinine (n = 18), 3HB (n = 18) and tryptase (n = 19) in almost all cases, showing that there is no passive shift with increasing post-mortem period above the thresholds in general. Whenever one measurement was above the post-mortem threshold, irrespective of being at T0, T1 or T2, then the marker level was at or above these cut-offs at T3.

The single cases illustrated in Fig. 3 showed underlying cardiac causes of death for seven out of nine cases with exceeding troponin T values. A considerable passing of the troponin T threshold value seemed to mostly appear only when the marker levels during onset of death were already increased.

### Quality control

**Figure 4:**
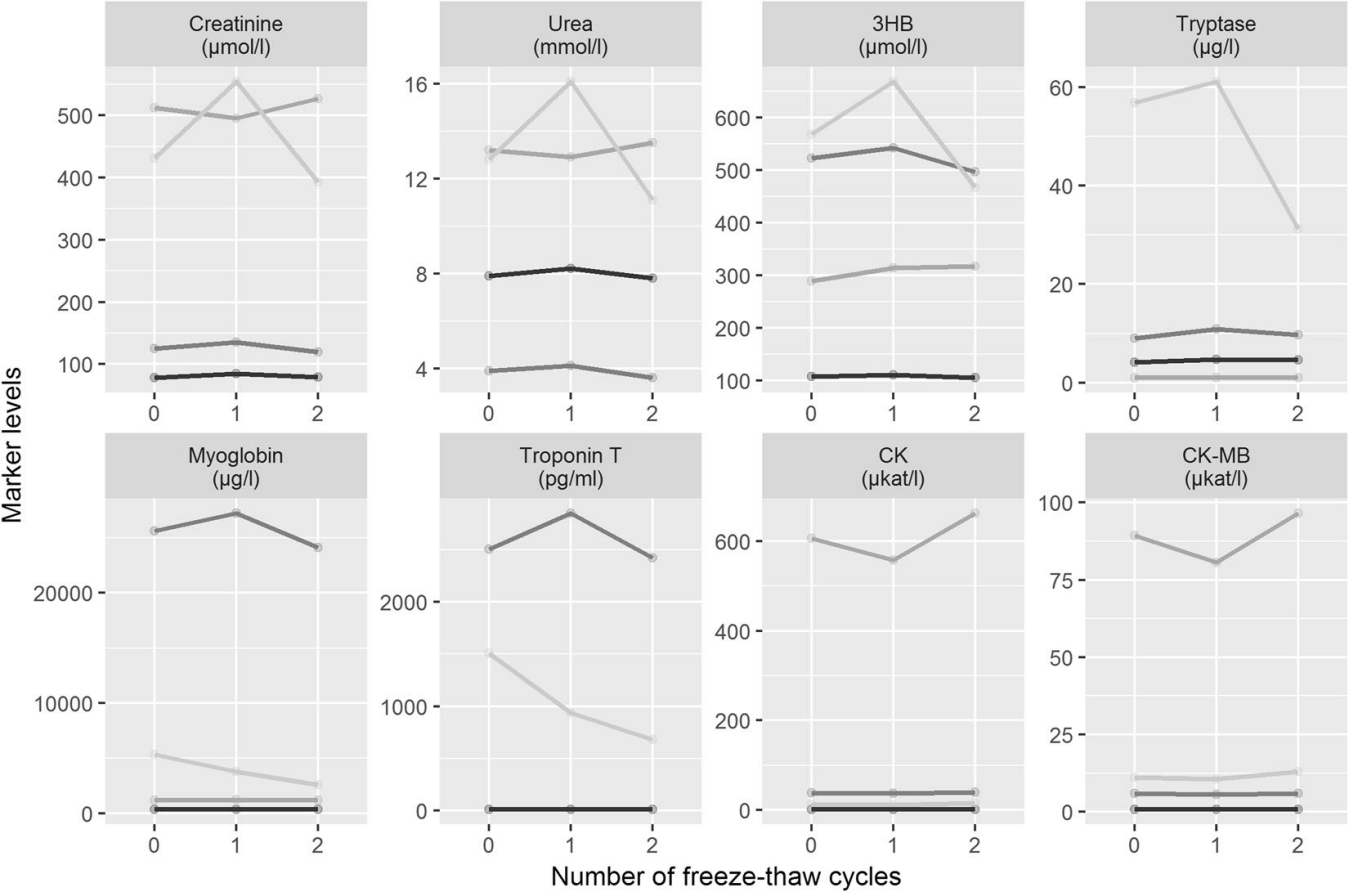
Laboratory results for three repeated freeze-thaw cycles, tested in the same four samples for all markers. 3HB, 3-β-hydroxybutyrate; CK, creatin kinase.

Freeze and thaw stability was tested to ascertain the possibility of repeated or delayed measurement of biochemical markers from serum samples, which are stored at −80 °C. Therefore, three freeze-thaw cycles (numbered as 0; 1; 2) were performed for each marker and at each point in time (T0-T3) among different cases. Figure 4 shows the changes of marker levels depending on the number of freeze-thaw cycles. Most analytes were stable regardless of the number of thawing cycles. Most markers showed at least one single sample with an elevated level after the first thawing compared to the initial value but with a subsequent decline beneath the initial value or vice versa. No single measurement presented levels above the post-mortem references when the initial concentration of the untreated sample was below these thresholds. Referring to the initial value measured, the maximum ranges after repeated thawing are shown in Supplemental Table 2.

**Figure 5:**
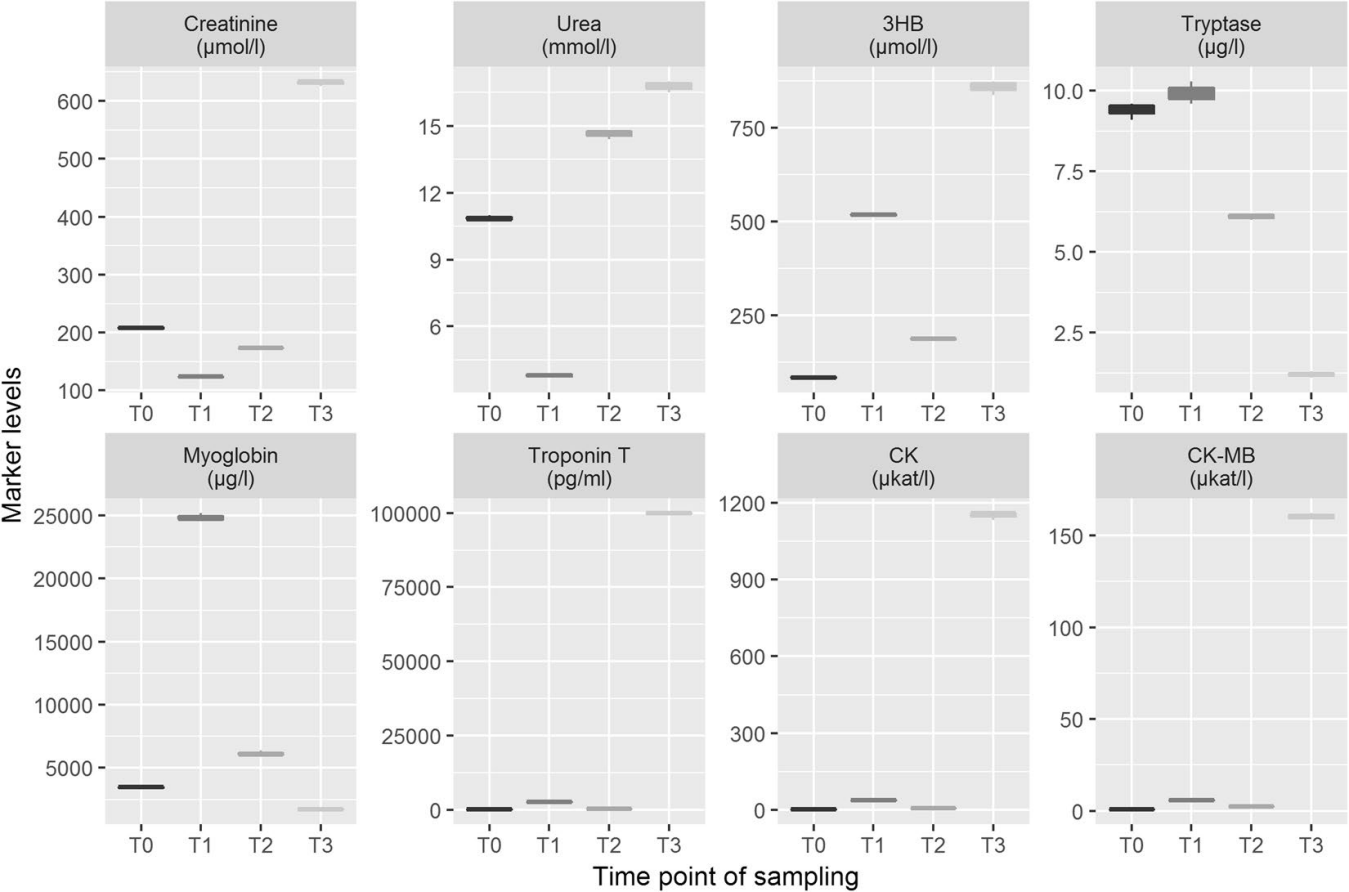
Results of triplicate measurement for all markers of the four samples selected (one sample representing one sampling time) to test the post-mortem intra-assay precision of the used kits. 3HB, 3-β-hydroxybutyrate; CK, creatin kinase.

The intra-assay precision of the post-mortem substrates was high for all markers tested. The numerical deviation of the investigated values never exceeded 10% (see Supplemental Table 2). When comparing the minute variance within the measurements with maximum deviations +5.5% as upper and −7.3% as lower range (illustrated in Fig. 5) with the absolute changes of the markers tested over increasing post-mortem interval (Fig. 2, Table 2), the changes in precision were negligible and within the definitions of precision according to the quality standards for medical laboratories of the German Medical Association.

**Figure 6:**
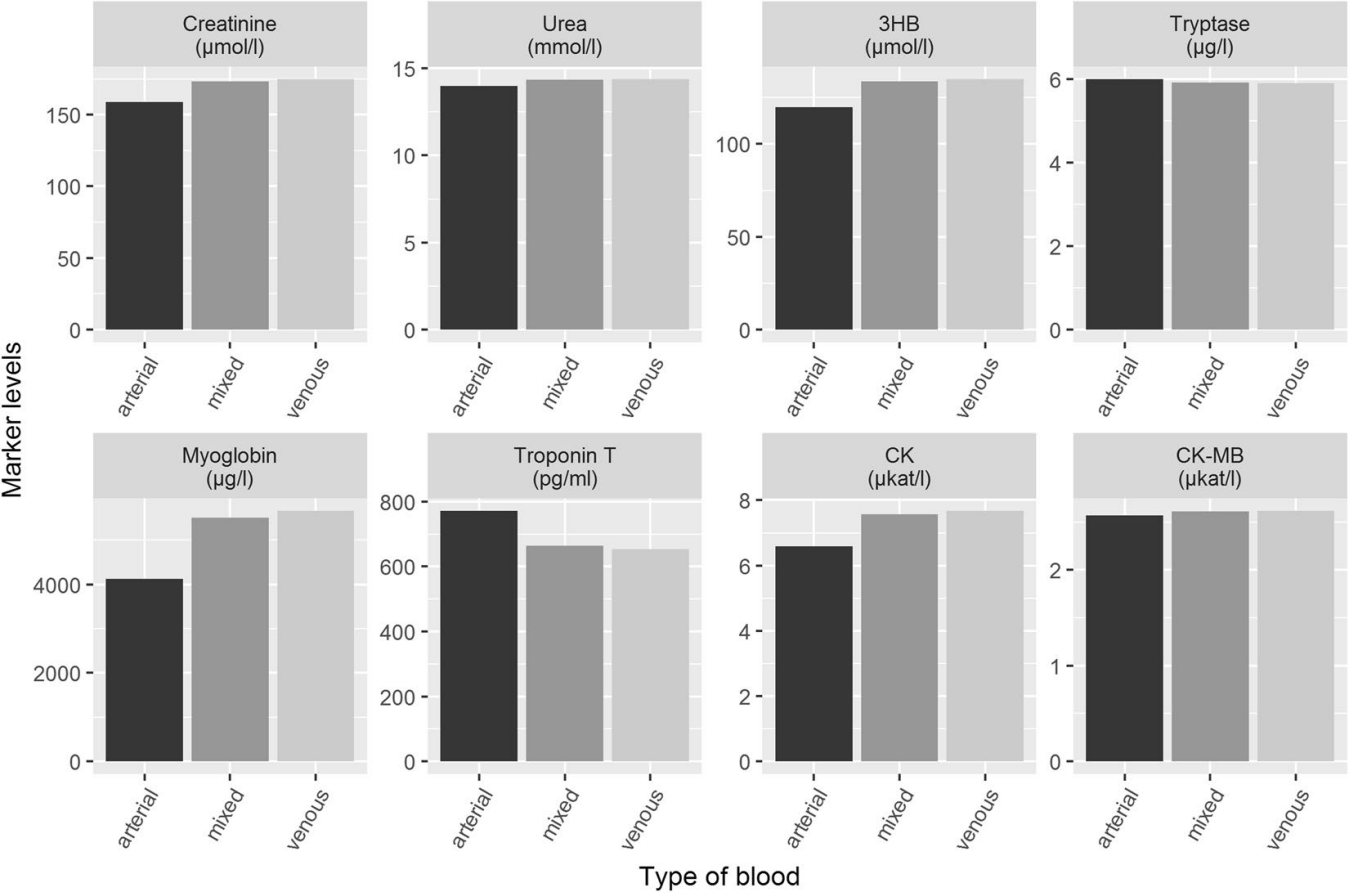
Differences of the marker levels when measured in arterial and venous blood samples. While some post-mortem sampling might be difficult and goes along with accidental arterial puncture, a mixture type (defined as 90% venous and 10% arterial) was illustrated also.

Finally, the values were checked for differences between the femoral arterial and venous samples. The relative deviation of the arterial value in relation to the venous value is presented in Supplemental Table 2, and was fluctuating around 10% depending on the marker tested with mostly higher levels in venous blood. Using these data, we determined to which extent an unintended ‘mixture’ of arterial and venous blood might affect the accuracy of the measurements (e.g. if an artery is accidentally punctured). We found no relevant changes when comparing mixed with pure arterial and pure venous blood (see Fig. 6).

## Discussion

The objective of this study was to determine the changes in different biochemical markers in blood serum after the onset of death. Our results indicate that the investigated markers underlie post-mortem changes with the tendency to rise proportionally to the post-mortem interval of 48 hours. However, creatinine, urea, 3HB and tryptase exceeded post-mortem cut-off values only in single cases and post-mortem urea and tryptase were even within clinical references ranges for most samples. All cardiac marker levels showed large increases post-mortem.

No marker had shown significant changes in the first two hours after death, therefore, the authors recommend that the collection of post-mortem blood samples as part of the external post-mortem examination should be done in this early time interval if the puncture of a blood vessel is legally possible. This offers the opportunity to significantly reduce the described influence of post-mortem changes within the blood samples. As a rule of thumb, the collection of blood should be carried out as soon as possible after death. The serum levels of all selected markers should ideally reflect functional or organic causes of death irrespective of the post-mortem interval. In forensic research, some other markers, especially electrolytes, were investigated to estimate the post-mortem interval biochemically, but reliable death time estimations are impossible in most cases using laboratory kits^30^.

Some authors described a slight increase in creatinine^14^, which is also shown in a mouse experiment^31^, and in urea^14,26,32^ after death, in line with our results. Nevertheless, both markers have been presented in literature as reliable post-mortem markers^1,3,14,31^ and post-mortem cut-offs are used for diagnosing lethal acute kidney failure^26^. These cut-offs seem to be sufficient, since no single value of urea was above its threshold, and only one single creatinine level increased above it during the post-mortem interval.

In previous studies, no correlation between ketone body concentration and the post-mortem interval was observed^27,33^. As 3HB is declared to be more stable than acetoacetate, especially regarding delayed measurement after storage, this is the marker of choice to determine the ketone status of a body^34^ and is used in daily forensic routine. Iten and Maier^27^ proposed a 3HB cut-off of 500 µmol/l and constituted that all levels under this should be assessed as ‘normal’ in post-mortem settings. Again, this cut-off was elevated only in two cases, and seemed to be useful in post-mortem biochemistry for investigating unselected cases.

In numerous existing publications it has been shown that the clinical reference value for tryptase cannot be used post-mortem, as tryptase levels after death are sometimes increased compared to samples of living patients even without underlying anaphylactic reactions^22,28,35–37^. Systematic development of this parameter enables the definition of a post-mortem cut-off value. Currently, the most common value is proposed to be 44.3 µg/l^28^. Further studies confirmed this limit^35^ or suggested similar reference values^22,37^. Only one T3 value of our cohort exceeded this post-mortem limit; the person had died from a cardiac cause of death as autopsy result without signs for any anaphylactic trigger.

Interestingly, evidence regarding tryptase development relative to the post-mortem interval is inconsistent. While some authors^36,37^ declared no tendency of increasing tryptase with post-mortem interval, others^35^ showed such dependency. The presented results indicate that there is indeed an increase with longer post-mortem interval, but almost all without consequences for the post-mortem cut-off promised. As there were no anaphylaxis cases in our cohort, we could not assess if there was a different post-mortem development in such special cases. Interestingly, Tse *et al*.^11^ presented a case of an anaphylactic death with initially increased values which subsequently decreased; another similar case was reported by Sravan *et al*.^12^. This may have been due to a degradation of the released hormones^12^ or just reflected the clinical development after anaphylaxis^38^.

Most troponin T studies showed a post-mortem increase, dependent on the post-mortem interval, at least after 48 h^39,40^. To the best of our knowledge, only one publication reported the post-mortem results of the same highly-sensitive troponin T assay from Roche Diagnostics and these authors have shown a general post-mortem increase in serum without relation to the underlying causes of death^29^. They tried to describe a post-mortem troponin T cut-off of 250 pg/ml^29^. A recent study showed comparable results when investigating cardiac causes of death^39^. Interestingly, the values of our given study often exceeded this previous threshold, but in most of the cases this was already true for the first post-mortem sample. This might be due to a heavy surge of troponin T from damaged cardiomyocytes or by prolonged CPR with secondary cardiac damage^41^. These attempts could have changed the initial values and distribution of biologic fluids, as it was stated that CPR disturbs agonal values deeply^42^ and shifts from central to peripheral blood^43^. Since 90% of our cohort got final chest compressions via CPR, we were not able to check for the influence of such rescue attempts by statistical methods. However, other authors stated no influence of CPR on post-mortem troponin T levels^39,44^.

There were a few attempts to measure post-mortem serum myoglobin and CK as well as CK-MB, for example with reference to electrical fatalities^13,23^. An increase in the values with the passage of post-mortem time was obvious for these (heart) muscle markers^23^ and a practically applicable cut-off value could have been determined for none of them^13,45^. An unspecified post-mortem increase of myoglobin dependent on the PMI could be determined decades ago, but without resulting recommendations^46^. These reports are in line with the presented results describing severe post-mortem alterations. Therefore, myoglobin, CK and CK-MB seem to be not useful in post-mortem biochemistry.

Costa *et al*.^25^ tried to reconstruct the development of 46 different markers (including creatinine, urea, CK and CK-MB) by simulating the exact post-mortem drop in temperature until reaching room temperature and measuring all parameters at 16 defined time points with the aim of establishing a formula to calculate the post-mortem interval. They found an increase in urea and CK and a stabilization of creatinine levels after 24 hours. However, their experiments were performed *in-vitro* with the serum of living patients. This may show the dependence on hemolysis of blood and change of temperatures after death. However, uncritical transmission on post-mortem samples has to be considered critically as many *in-situ* influences could not get captured *in-vitro*, such as passive diffusion, functional loss of tissue and advanced autolytic processes. The present results systematically show increasing rates of hemolysis and lipemia with longer post-mortem interval.

All tested markers are equipped with very high intra-assay precisions even for post-mortem samples. This allows economic laboratory processes (single measurement per sample) for every marker. The freeze-thaw stability of the marker appears to be acceptable in light of the overall changes along the post-mortem interval. This allows a centralization of post-mortem biochemical trials in experienced labors on the one hand and a freeze-storage shipping of serum samples, if that should become necessary.

In some cases, femoral blood samples are only collected by scratching the legs to the groins during autopsy in daily routine and not by puncture of vessels, as was done here. We could show that a potential mixture of arterial and venous blood does not relevantly affect the post-mortem biochemical measurements.

Further studies should investigate the post-mortem *in-situ* behavior of other biochemical markers which are used in forensic research and routine, for example biomarkers of traumatic cerebral damage^47,48^ or acute inflammation^49^ and sepsis^1,2,9,10^.

In this prospective study, a relatively small cohort of 20 cases was included given the multiple numbers of samples taken from each of the deceased. This limited number was accepted to avoid long-term freeze storage of single samples as little is known about marker changes after longer storing periods in forensic^50^ as well as clinical samples^51^. Though intra-individual post-mortem changes could be presented, this number was not acceptable to make a sufficient point in new cut-off values after death or reliable regression analysis for mathematical determination of initial values, therefore further data is required. Additionally, further studies should investigate the problem of long-term freeze storage in different conditions.

No declaration regarding usefulness of the markers as surrogate of different causes of death can be given thoroughly due to the small number of cases and the heterogeneity of the individual cases, as we did not investigate any case or control subgroup. Previous studies showed the dependence of markers on different sampling sites – this is why the presented values only apply to serum measurements from femoral venous blood^52–54^. According to Uemura *et al*.^52^, the femoral venous blood should be regarded as gold standard for post-mortem biochemical trials.

Time dependency plays the most important role in reliable post-mortem biochemistry in our view. Therefore, the results of intra-individual stability presented can only represent the early post-mortem period within two days of death. An influence of temperature on the markers has already been shown before^25^. The bodies examined in this study were stored in a cold storage cell at 4 °C. As most of the corpses in forensic medicine succumb different changes in different temperatures, the change of the markers in forensic practice might slightly deviate. It was stated that relevant hemolysis may take 48 hours if the bodies were sufficiently cooled^32^, so hemolytic processes were slowed down in this study as much as possible.

Notwithstanding the illustrated limitations, we could document systematic post-mortem changes *in-situ* of all investigated markers in different intensities. We conclude that an uncritical interpretation of post-mortem values may lead to false positive results and that it is essential to know the post-mortem interval until the blood sampling. Post-mortem biochemistry can never be the only instrument of a forensic pathologist in a post-mortem examination but has great potential for daily routine in investigating sudden unexpected deaths with no or minute ante-mortem data.

## Methods

### Sample collection

This research study has been approved by the ethics committee of the medical faculty of the University of Leipzig, Germany (local number 388/15-ek) and in any individual case, the next of kin of the deceased granted informed and written consent. All methods were carried out in accordance with the relevant guidelines and regulations.

In this prospective monocentric study covering a 12 months period, post-mortem blood samples were collected serially from 20 deceased (sampled over a period of ten months between first and last individual included) and measurements of carefully selected biomarkers were performed.

Blood samples were collected at four predetermined post-mortem time points with two filled serum tubes each, whereas the first sample was taken directly after occurrence of death (=T0). To receive the possibility of getting a blood sample exactly after a patient died, we only included patients that arrived with ongoing CPR in the Emergency Department (ED) of the University Hospital of Leipzig or who died suddenly in the resuscitation room without final CPR because of known patient’s provision.

The definite time of death was defined through termination of CPR at the hands of the responsible emergency physician or through the observation of circulatory arrest in patients who refused CPR. Instantly, the ED physician performed the first blood sample collection by a sterile puncture of peripheral vessels. The blood samples were immediately cooled in a refrigerator at 4 °C.

The second sample (=T1) from the femoral veins was aseptically collected with sterile syringes by a forensic pathologist during the external examination of the corpse using the typical post-mortem puncture technique^55^ (supported by ultrasound assistance in one case). Without delay, the cooled T0 and T1 samples were centrifuged for 10 minutes at 5,000 rpm with subsequent separation of the supernatant and the aliquots were stored deep-frozen at −80 °C.

The third (=T2) and fourth (=T3) blood samples were taken in the Institute of Legal Medicine through external femoral venous puncture as described above (for all T2, supported by ultrasound assistance in three cases and for n = 17 of T3 samples) respectively during autopsy (only T3, n = 3) and immediately centrifuged, separated and stored as already described.

For some of the markers, one additional sample was available due to ante-mortem blood sampling during CPR by the emergency physician. When existent, the changes between this pre-sample and the first post-mortem sample (=T0) were also evaluated.

Exemplarily, blood samples of the cohort were used to test thawing stability by performing three repeated freeze-thaw procedures. The chosen example tubes (n = 4 individuals) were thawed to room temperature for four hours with a subsequent freezing cycle of 24 hours.

Further, we tested the intra-assay precision for the post-mortem substrates by triplicate measurement of exemplar samples (n = 4) of all four time points of sampling.

Finally, one blood sample was collected per ultrasound-assisted arterial groin puncture to compare the biochemical results of arterial blood with the ones out of the venous blood system post-mortem to check for arterial-venous differences in post-mortem marker levels.

### Laboratory analyses

The serum samples were measured in batch at the Leipzig University Hospital’s central laboratory for diagnostics (Institute of Laboratory Medicine). The laboratory is accredited by ISO 15189 and all analyzes were performed based on routine calibration according to the quality standards for medical laboratories of the German Medical Association. All measurements were performed on standard automated clinical chemistry analyzers (Cobas 8000, Roche Diagnostics, Mannheim, Germany) using *in-vitro* diagnostics certified assays for creatinine (Roche, ref. 05168589190; photometry, enzymatic reaction; measuring range 5–2,700 μmol/l), urea (Roche, ref. 05171873190; photometry, kinetic assay; measuring range 0.5–40 mmol/l), 3HB (Wako Chemicals, Neuss, Germany, ref. 417–73501; photometry, cyclic enzymatic method; measuring range 3–1,000 μmol/l), mast cell tryptase (Phadia, Uppsala, Sweden, ref. CAP250, fluorimetric enzyme-linked immunoassay; measuring range 1–200 μg/l), myoglobin (Roche, ref. 07027538190, ElectroChemiLuminescence immunoassay; measuring range 21–3,000 μg/l), highly-sensitive troponin T (Roche, ref. 07028075190, ElectroChemiLuminescence immunoassay; measuring range 3–10,000 pg/ml), CK (Roche, ref. 07190794190, photometry, UV test; measuring range 0.12–33.4 μkat/l) and CK-MB (Roche, ref. 07190808190, immunologic UV test; measuring range 0.05–33.4 μkat/l).

If test results exceeded the measuring range (defined by the manufacturer), serum samples were diluted according to the manufacturers maximally allowed dilution factor (1:10) and re-measured. Test results still exceeding the measuring range after dilution were assigned the maximum quantitative value. This was applied to 17 samples of myoglobin measurement (30,000 μg/l) and one sample of troponin T measurement (100,000 pg/ml). Only three single measurements of the whole batch were below the limits of detection according to the manufacturer and were assigned to this specific minimum value (tryptase, n = 2; troponin T, n = 1).

Laboratory analysis also included the automated measurements of three serum interference indices using a Cobas c701 analyzer (Roche) to check for quality purposes (hemolysis index, lipemia index, icterus index). These indices are calculated by absorbance measurements with different bichromatic wavelength pairs of saline diluted samples as semi-quantitative representation of levels of hemolysis (measured at 600/570 nm; correlating with the hemoglobin concentration in the sample), lipemia (measured at 700/660 nm; as estimation of sample turbidity) or icterus (measured at 505/480 nm; correlating with the bilirubin level in the sample).

All investigators were entirely blinded to all patients’ data while carrying out the assays.

### Statistical analyses

Data analysis was conducted using the statistical software R (version 3.4.0, 2017; open source) and Microsoft Excel (2016; Redmond, WA, USA).

First, we checked the data for normality using Shapiro-Wilk tests and quantile-quantile plots and transformed the data, applying the natural logarithm if necessary. Then, the correlation between different sample indices and marker levels was tested within a linear mixed regression model thereby modelling individuals as random intercept term. Resulting p values were corrected for multiple testing using the Benjamin-Hochberg procedure. Afterwards, the different time intervals were tested per marker for statistically significant differences using non-parametric Friedman tests. Again, multiple testing for the markers was done using the Benjamini-Hochberg procedure. For statistically significant results, *post-hoc* tests investigating significance of time intervals according to Conover’s test were done. Thereby, *post-hoc* tests were corrected by a Bonferroni adjustment to avoid type I error accumulation. Adjusted p values of 0.05 or less were considered as statistically significant.

## Electronic supplementary material

Dataset 1

## Data Availability

All data generated or analyzed during this study are included in this published article and its supplementary information files.

## References

[1] Palmiere C, Mangin P (2012). Post-mortem chemistry update part I. Int. J. Legal Med..

[2] Palmiere C, Mangin P (2012). Post-mortem chemistry update part II. Int. J. Legal Med..

[3] Coe J (1993). Post-mortem chemistry update. Emphasis on forensic application. Am. J. Forensic Med. Patho..

[4] Luna A (2009). Is post-mortem biochemistry really useful? Why is it not widely used in forensic pathology?. Legal Med..

[5] Maeda H, Zhu B-L, Ishikawa T, Quan L, Michiue T (2009). Significance of post-mortem biochemistry in determining the cause of death. Legal Med..

[6] Belsey SL, Flanagan RJ (2016). Post-mortem biochemistry. Current applications. J. Forensic Leg. Med..

[7] Madea B (2005). Is there recent progress in the estimation of the post-mortem interval by means of thanatochemistry?. Forensic Sci. Int..

[8] Reichelt U, Jung R, Nierhaus A, Tsokos M (2005). Serial monitoring of interleukin-1beta, soluble interleukin-2 receptor and lipopolysaccharide binding protein levels after death. A comparative evaluation of potential post-mortem markers of sepsis. Int. J. Legal Med..

[9] Tsokos M, Reichelt U, Jung R, Nierhaus A, Püschel K (2001). Interleukin-6 and C-reactive protein serum levels in sepsis-related fatalities during the early post-mortem period. Forensic Sci. Int..

[10] Tsokos M, Reichelt U, Nierhaus A, Püschel K (2001). Serum procalcitonin (PCT). A valuable biochemical parameter for the post-mortem diagnosis of sepsis. Int. J. Legal Med..

[11] Tse R, Garland J, Ahn Y (2018). Decline in 2 serial post-mortem tryptase measurements beyond 72 hours after death in an antibiotic-related anaphylactic death. Am. J. Forensic Med. Pathol..

[12] Sravan A, Tse R, Cala AD (2015). A Decline in 2 consecutive post-mortem serum tryptase levels in an anaphylactic death. Am. J. Forensic Med. Pathol..

[13] Fieguth A, Schumann G, Tröger HD, Kleemann WJ (1999). The effect of lethal electrical shock on post-mortem serum myoglobin concentrations. Forensic Sci. Int..

[14] Zhu BL (2002). Post-mortem serum uric acid and creatinine levels in relation to the causes of death. Forensic Sci. Int..

[15] Zhu B-L (2007). Differences in post-mortem urea nitrogen, creatinine and uric acid levels between blood and pericardial fluid in acute death. Legal Med..

[16] Maeda H (2008). Post-mortem serum nitrogen compounds and C-reactive protein levels with special regard to investigation of fatal hyperthermia. Forensic Sci. Med. Pathol..

[17] Laffel L (1999). Ketone bodies. A review of physiology, pathophysiology and application of monitoring to diabetes. Diabetes Metab. Res. Rev..

[18] Elliott S, Smith C, Cassidy D (2010). The post-mortem relationship between beta-hydroxybutyrate (BHB), acetone and ethanol in ketoacidosis. Forensic Sci. Int..

[19] Palmiere C (2016). Post-mortem biochemistry in suspected starvation-induced ketoacidosis. J. Forensic Leg. Med..

[20] Kanetake J (2005). The relationship of a high level of serum beta-hydroxybutyrate to cause of death. Legal Med..

[21] Payne V, Kam PCA (2004). Mast cell tryptase. A review of its physiology and clinical significance. Anaesthesia.

[22] Tse R (2018). Post mortem tryptase cut-off level for anaphylactic death. Forensic Sci. Int..

[23] Püschel K, Lockemann U, Bartel J (1995). Post-mortem investigation of serum myoglobin levels with special reference to electrical fatalities. Forensic Sci. Int..

[24] Ellingsen CL, Hetland Ø (2004). Serum concentrations of cardiac troponin T in sudden death. Am. J. Forensic Med. Pathol..

[25] Costa I (2015). Promising blood-derived biomarkers for estimation of the post-mortem interval. Toxicol. Res..

[26] Kernbach-Wighton G (2009). Diagnostic problems with functional causes of death. Analytical approaches and procedures. Legal Med..

[27] Iten PX, Meier M (2000). Beta-hydroxybutyric acid–an indicator for an alcoholic ketoacidosis as cause of death in deceased alcohol abusers. J. Forensic Sci..

[28] Edston E, Eriksson O, van Hage M (2007). Mast cell tryptase in post-mortem serum – reference values and confounders. Int. J. Legal Med..

[29] Gonzalez-Herrera L, Valenzuela A, Ramos V, Blazquez A, Villanueva E (2016). Cardiac troponin T determination by a highly sensitive assay in post-mortem serum and pericardial fluid. Forensic Sci. Med. Pathol..

[30] Ortmann J, Markwerth P, Madea B (2016). Precision of estimating the time since death by vitreous potassium - comparison of 5 different equations. Forensic Sci. Int..

[31] Nishida A (2015). Blood creatinine level in post-mortem cases. Sci. Justice.

[32] Forrest AR (1993). Obtaining samples at post mortem examination for toxicological and biochemical analyses. J. Clin. Pathol..

[33] Thomsen JL, Felby S, Theilade P, Nielsen E (1995). Alcoholic ketoacidosis as a cause of death in forensic cases. Forensic Sci. Int..

[34] Fritzsche I, Bührdel P, Melcher R, Böhme HJ (2001). Stability of ketone bodies in serum in dependence on storage time and storage temperature. Clin. Lab..

[35] Horn KD, Halsey JF, Zumwalt RE (2004). Utilization of serum tryptase and immunoglobulin E assay in the post-mortem diagnosis of anaphylaxis. Am. J. Forensic Med. Pathol..

[36] Mayer DE (2011). Usefulness of post mortem determination of serum tryptase, histamine and diamine oxidase in the diagnosis of fatal anaphylaxis. Forensic Sci. Int..

[37] Xiao N (2017). Post-mortem serum tryptase levels with special regard to acute cardiac deaths. J. Forensic Sci..

[38] Palmiere, C. Decline in post-mortem serum tryptase levels in anaphylactic deaths. *Am. J. Forensic Med. Pathol*. **38**, 274–275 (2017). 10.1097/PAF.0000000000000320 28691949

[39] Palmiere C (2018). Cardiac troponins and NT-proBNP in the forensic setting: Overview of sampling site, post-mortem interval, cardiopulmonary resuscitation, and review of the literature. Forensic Sci. Int..

[40] Remmer S, Kuudeberg A, Tõnisson M, Lepik D, Väli M (2013). Cardiac troponin T in forensic autopsy cases. Forensic Sci. Int..

[41] Dreßler J, Felscher D, Koch R, Müller E (1998). Troponin T in legal medicine. Lancet.

[42] Madea B, Musshoff F (2007). Post-mortem biochemistry. Forensic Sci. Int..

[43] Flanagan RJ (2017). Post-mortem biochemistry and toxicology. Arab. J. For. Sci. For. Med..

[44] Chen J-H (2015). Cardiac biomarkers in blood, and pericardial and cerebrospinal fluids of forensic autopsy cases. A reassessment with special regard to post-mortem interval. Legal Med..

[45] Zhu BL (2007). Post-mortem cardiac troponin I and creatine kinase MB levels in the blood and pericardial fluid as markers of myocardial damage in medicolegal autopsy. Legal Med..

[46] Suzuki T, Kashimura S, Umetsu K (1983). Post-mortem permeation of myoglobin into the blood. Z. Rechtsmed..

[47] Sieber M, Dreßler J, Franke H, Pohlers D, Ondruschka B (2018). Post-mortem biochemistry of NSE and S100B: A supplemental tool for detecting a lethal traumatic brain injury?. J. Forensic Leg. Med..

[48] Ondruschka B, Schuch S, Pohlers D, Franke H, Dreßler J (2018). Acute phase response after fatal traumatic brain injury. Int. J. Legal Med..

[49] Ondruschka, B., Sieber, M., Kirsten, H., Franke, H. & Dreßler, J. Measurement of cerebral biomarkers proving traumatic brain injuries in post-mortem body fluids. *J. Neurotrauma* in press (2018). 10.1089/neu.2017.5441 29732941

[50] Hess C, Krueger L, Unger M, Madea B (2017). Freeze-thaw stability and long-term stability of 84 synthetic cannabinoids in serum. Drug Test. Anal..

[51] Cuhadar S, Koseoglu M, Atay A, Dirican A (2013). The effect of storage time and freeze-thaw cycles on the stability of serum samples. Biochem. Med..

[52] Uemura K (2008). Biochemical blood markers and sampling sites in forensic autopsy. J. Forensic Leg. Med..

[53] Felby S, Nielsen E, Thomsen JL (2008). The post-mortem distribution of ketone bodies between blood, vitreous humor, spinal fluid, and urine. Forensic Sci. Med. Pathol..

[54] Palmiere C, Mangin P (2015). Urea nitrogen, creatinine, and uric acid levels in post-mortem serum, vitreous humor, and pericardial fluid. Int. J. Legal Med..

[55] Huckenbeck, W. & Bonte, W. Blood sampling on corpses in *Handb. Gerichtl. Med*. (eds Madea, B. & Brinkmann, B.) 469–473 (Springer, 2003).

