# Supplementary material for: Intra-individual alterations of serum markers routinely used in forensic pathology depending on increasing post-mortem interval
Source: Sci Rep. 2018 Aug 24;8:12811. doi: 10.1038/s41598-018-31252-5 (PMC6109050; doi:10.1038/s41598-018-31252-5)
Supplement: Supplementary file 1 — Dataset 1 [file 41598_2018_31252_MOESM1_ESM.pdf]

# Intra-individual alterations of serum markers routinely used in forensic pathology depending on increasing post-mortem interval

Lina Woydt[1,#] , Michael Bernhard[2,3,#], Holger Kirsten[4,5], Ralph Burkhardt[6], Niels Hammer[7-9], André Gries[2], Jan Dreßler[1], Benjamin Ondruschka[1,*]

[1] Institute of Legal Medicine, Medical Faculty University of Leipzig, Leipzig, Germany
[2] Emergency Department, University Hospital of Leipzig, Leipzig, Germany
[3] Emergency Department, Heinrich Heine University Duesseldorf, Duesseldorf, Germany
[4] Institute for Medical Informatics, Statistics and Epidemiology, University of Leipzig, Leipzig, Germany
[5] LIFE Research Center for Civilization Diseases, University of Leipzig, Leipzig, Germany
[6] Institute of Laboratory Medicine, Clinical Chemistry and Molecular Diagnostics, University of Leipzig, Leipzig, Germany
[7] Department of Anatomy, University of Otago, Dunedin, New Zealand
[8] Department of Orthopedic and Trauma Surgery, University Hospital of Leipzig, Leipzig, Germany
[9] Fraunhofer IWU, Dresden, Germany

# Both authors contributed equally to this work.

[*] **Corresponding Author:** Dr. med. Benjamin Ondruschka
Medical Faculty University of Leipzig
Institute of Legal Medicine
Johannisallee 28
D-04103 Leipzig, Germany
phone: +49 (0) 0341-9715152
fax: +49 (0) 341-9715109
mail: benjamin.ondruschka@medizin.uni-leipzig.de

## Electronic supplementary material

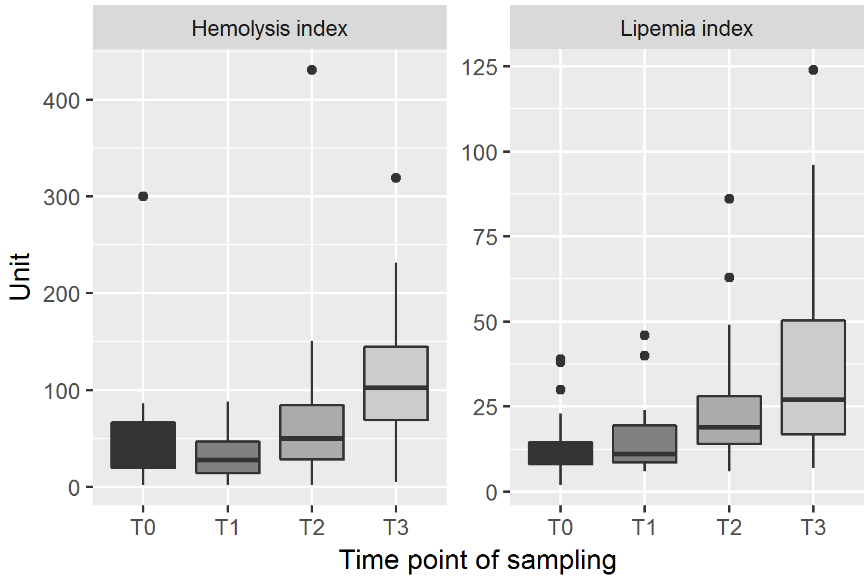


**Supplemental figure 1:** Changes of hemolysis index (left) and lipemia index (right) according to the different sampling time points reflecting increasing post-mortem interval.

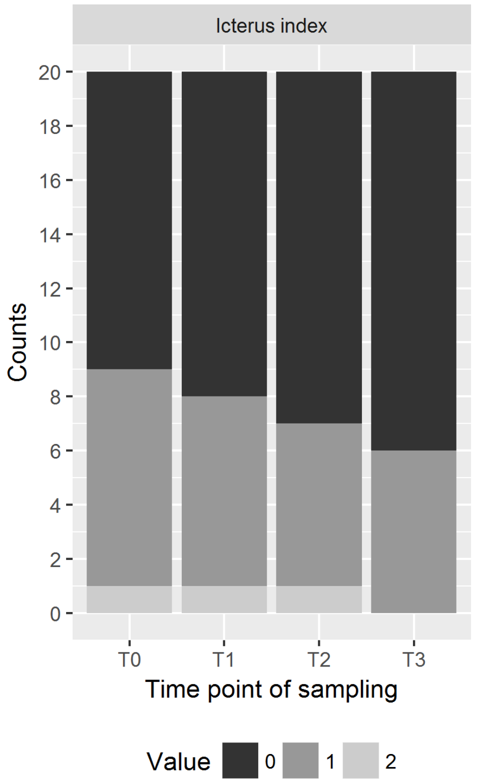


**Supplemental figure 2:** Minute changes of icterus index according to the different time points of sampling reflecting increasing post-mortem interval.

| Marker | Upper clinical reference | Post-mortem cut-off values | |
|---|---|---|---|
| | | Value | Citation |
| Creatinine (in µmol/l) | 104 | 353.6 | Kernbach-Wighton [26] |
| Urea (in mmol/l) | 8.3 | 33.4 | Kernbach-Wighton [26] |
| 3HB (in µmol/l) | 74 | 500 | Iten & Meier [27] |
| Tryptase (in µg/l) | 11.4 | 44.3 | Edston et al. [28] |
| Myoglobin (in µg/l) | 72 | *not reported* | *not available* |
| Troponin T (in pg/ml) | 14 | 250 | Gonzalez-Herrera et al. [29] |
| CK (in µkat/l) | 2.9 | *not reported* | *not available* |
| CK-MB (in µkat/l) | 0.4 | *not reported* | *not available* |

**Supplemental table 1:** Comparison between upper clinical reference values presented by the manufacturer (Roche Diagnostics, Mannheim, Germany) and post-mortem threshold values, linked to one main reference.

| Quality control | Creatinine | Urea | 3HB | Tryptase | Myoglobin | Troponin T | CK | CK-MB |
|---|---|---|---|---|---|---|---|---|
| **Freeze-thaw stability** | | | | | | | | |
| Upper range | +28.8% | +25.8% | +17.6% | +21.1% | +6.4% | +13.9% | +51.8% | +16.9% |
| Lower range | -8.8% | -13.3% | -17.9% | -45.2% | -51.0% | -54.9% | -8.0% | -9.7% |
| **Triplicate** | | | | | | | | |
| Upper range | +1.3% | +0.3% | +4.4% | +5.5% | +5.3% | +1.1% | +2.4% | +2.6% |
| Lower range | -2.5% | 0.0% | -3.2% | -7.3% | -2.3% | -6.5% | -3.4% | -2.6% |
| **Arterial-venous deviation** | | | | | | | | |
| Maximum relative | 9.1% | 2.8% | 11.3% | 1.7% | 27.3% | 18.3% | 14.3% | 1.9% |

**Supplemental table 2:** Numerical details of quality control checks.
